# Hemolytic anemia due to pyruvate kinase deficiency coexistent with the alpha thalassemia trait and chronic myeloid leukemia

**DOI:** 10.1016/j.htct.2024.04.130

**Published:** 2024-09-12

**Authors:** Nirmal Shrestha, Harshit Khurana, Renjith Verghese, Yogendra Mishra

**Affiliations:** Armed Forces Medical College, Pune, India

## Introduction

Pyruvate kinase (PK) deficiency is the most frequent red cell enzyme deficiency that causes hemolysis with a prevalence of 5:100,000 in most populations.^1^ Association of PK deficiency with hemoglobinopathies is rare with few reported cases.^2^ Moreover, its association with chronic myeloid leukemia (CML) has not been reported in the literature yet. Hence, we report an extremely rare presentation of PK deficiency hemolytic anemia with alpha thalassemia trait and CML.

## Case report

This patient is a 25-year-old male, resident of western India. He had presented with jaundice during the neonatal period and was treated with phototherapy. At three months of age, he presented as a failure to thrive child. On investigation, he was found to have low hemoglobin (Hb: range: 4–5 g/dL) along with increased indirect bilirubin and lactate dehydrogenase (LDH). He was put on blood transfusions every 3–4 weeks for the maintenance of Hb levels.

At 15 years of age, he was evaluated for hemolytic anemia. High-performance liquid chromatography was negative for beta-thalassemia with Hb A_2_ of 2.8%, Hb F of 1.4% and no other abnormal Hb. Workup for red cell enzyme assay for glucose-6-phosphate dehydrogenase, PK, glucose phosphate isomerase, hexokinase, glyceraldehyde 3-phosphate dehydrogenase, pyrimidine 5′-nucleotidase ratio, and unstable Hb (heat stability test and isopropanol test) were normal. Direct coombs test/indirect coombs test and osmotic fragility test were also normal (Table 1 presents a summary of relevant investigations). Despite evaluations, the etiology for hemolytic anemia was obscure. He underwent a splenectomy at 16 years of age because of severe hemolytic anemia and he was on minimal (3–4) transfusions per year thereafter.

**Table 1:** Summary of relevant investigations.

Investigation			Results			Normal value		
Hemoglobin -(g/dL)			Age		Value	13.5 −18		
			03 months		4.5			
			15 years		3.5			
			20 years		6.6			
			25 years		11			
			25 years		8.5			
Total direct bilirubin - (mg/dL)			15 years		5.6/2.4	Direct bilirubin: 0.2–1.0 Indirect bilirubin: 00–0.2		
			20 years		4.1/0.4			
			25 years		1.4/0.7			
Lactate dehydrogenase - IU/L			2338			85–227		
Glucose 6 phosphate Dehydrogenase - IU/g Hb			13.14			6.5 to 14.0		
Pyruvate kinase - IU/g Hb			14.15			9.0 to 14.0		
Glucose phosphate isomerase - IU/g Hb			74.10			45 to 75.0		
Hexokinase - IU/g Hb			2.3			0.8 to 2.0		
Glyceraldehyde-3-phosphate dehydrogenase - IU/g Hb			281.6			250.0 to 350.0		
6-Phosphogluconate dehydrogenase - IU/g Hb			12.15			6.0 to 13.0		
Pyrimidine 5′-nucleotidase ration - IU/g Hb			3.051			2.5 to 3.5		
Flow cytometric analysis of red cell using Eosin-5-maleimide in mean channel fluorescence (MCF) units			1183.83			900 to 1300		
High performance liquid chromatography -%			Normal Hb A2: 2.8% Hb F: 1.4% No other abnormal hemoglobin			Hb A2 <3.5 Hb *F* < 2.0		
Unstable hemoglobin (Heat stability test and Isopropanol test)			Negative					
Direct Coombs test			Negative					
Indirect Coombs test			Negative					
Osmotic fragility test			Negative					
BCR ABL gene rearrangement test by Real Time PCR			Major translocation P210 (e13a2, e14a2, major)					
Mutational analysis for thalassemia			Negative for beta thalassemia Heterozygous deletion detected in thalassemia alpha mutation analysis (-α 3.7α/αα or αα/-α3.7α)					
Next generation clinical exome sequencing	‘Heterozygous’ variants were detected in PKLR gene (compound heterozygous) **Variant Table**							
	**Gene**	**Genomic location**		**Variant**	**Exon**	**Type**	**Zygosity**	**Phenotype group**
	PKLR	chr1: 155,261,709		NM_000298.5: c.1456C>T; p.Arg486Trp	10	Missense	Heterozygous	Pyruvate kinase deficiency
	PKLR	chr1: 155,263,320		NM_000298.5: c.1178A>*G*; p.Asn393Ser	8	Missense	Heterozygous	Pyruvate kinase deficiency

At 25 years of age, he presented with epistaxis. On evaluation, his Hb was 11 g/dL, total leukocyte count (TLC) 527,000 /mm^3^ and a peripheral blood smear was suggestive of myeloproliferative neoplasm, likely CML – chronic phase. (Figure 2 shows the peripheral blood smear with blasts suggestive of CML). He was positive for the breakpoint cluster region-Abelson oncogene (BCR-ABL) rearrangement with a major translocation (P210 (e13a2, e14a2) and initiated imatinib treatment. After three months, his Hb level had dropped to 8.5 g/dL. Mutational analysis revealed the alpha thalassemia mutation-heterozygous deletion (-α 3.7α/αα or αα/-α3.7α). No Beta thalassemia mutation was detected. Furthermore, next generation clinical exome sequencing detected ‘Heterozygous’ variants in the PKLR gene (compound heterozygous, viz. NM_000298.5:c.1456C>T;p.Arg486Trp and NM_000298.5: c.1178A>*G*; p.Asn393Ser) Table 1 .

**Figure 1:**
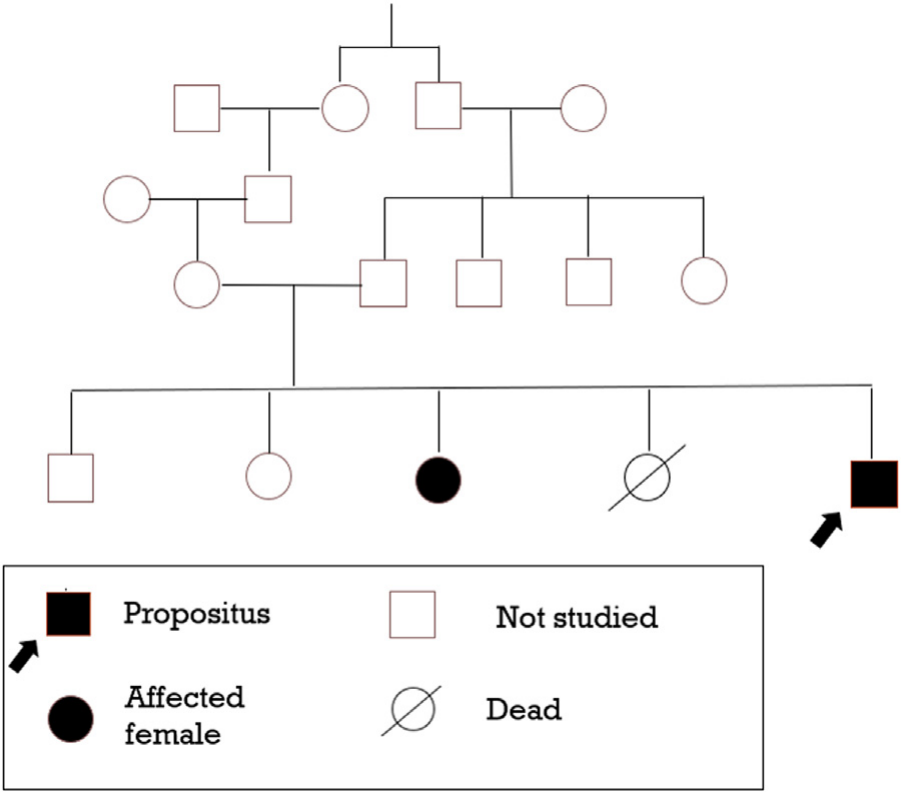
Graphical representation of family studies.

The parents of the patient are related first cousins. He has an elder sister who has a similar clinical profile (transfusion-dependent hemolytic anemia, indirect hyperbilirubinemia, and splenectomy at 18 years of age). Moreover, he had a sibling who died at 3.5 years of age who had a history of neonatal jaundice and kernicterus. Figure 1 (A graphical representation of family studies)

**Figure 2:**
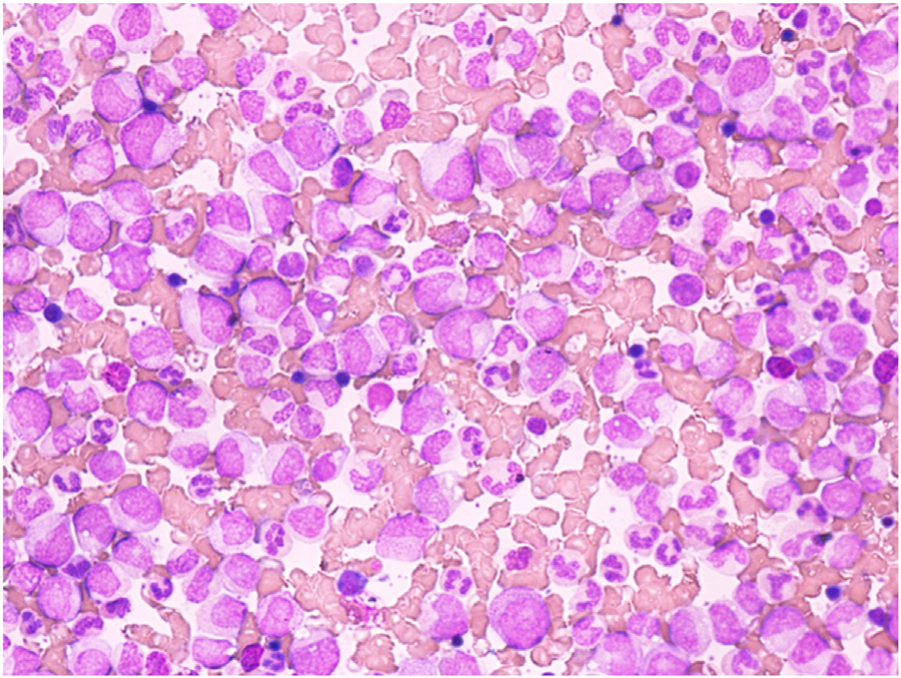
Peripheral blood smear showing leukocytosis with myelocyte bulge, blasts, basophils, promyelocytes, metamyelocytes, bands, eosinophils and monocytes suggestive of chronic myeloid leukemia likely the chronic phase.

Final Diagnosis:

1. Congenital non-spherocytic coombs negative hemolytic anemia due to red blood cell enzyme deficiency - PK
2. CML - chronic phase
3. Alpha thalassemia trait

He was managed with tyrosine kinase inhibitors and component support to maintain Hb >8 g/dL.

## Discussion

PK deficiency is an autosomal recessive disorder. The condition manifests phenotypically as hemolytic anemia in persons with compound heterozygous and homozygous variants. The severity of anemia varies greatly, from nonimmune hydrops fetalis or life-threatening neonatal anemia to extremely mild anemia or fully compensated hemolysis.^3^ Typically, siblings of the same family have similar disease severity.^1^ Our patient is heterozygous for an abnormal PK gene which was also found in his sibling. In the majority of reference laboratories, a standardized assay is still the preferred technique for determining PK activity. False normal levels may result due to recent transfusions or incomplete removal of platelets, white blood cells or reticulocytosis.^3^ This patient had normal pyruvate enzyme activity which may be a false negative result.

This patient underwent high performance liquid chromatography at the age of 15 years which was negative for beta thalassemia. Although he had been receiving frequent blood transfusions, the negative result was supported by the mutational analysis for thalassemia done later. Mutational analysis revealed a heterozygous alpha thalassemia mutation. The degree of hemolysis, anemia and blood transfusion requirement could not be explained by the heterozygous alpha thalassemia alone. Therefore, next generation clinical exome sequencing was carried out to identify the underlying disorder. It revealed compound heterozygous mutations, viz. NM_000298.5:c.1456C>T;p.Arg486Trp and NM_000298.5: c.1178A>*G*; p.Asn393Ser in the PKLR gene. The combination of both heterozygous alpha thalassemia and compound heterozygous PK deficiency could well explain the phenotypic presentation in the patient.

The association of PK deficiency with hemoglobinopathies has been reported in the literature.^2^^,^^4^ However, it is extremely uncommon for hereditary PK deficiency to progress into leukemias and only one case of acute monocytic leukemia with PK deficiency^5^ and one case of chronic myelomonocytic leukemia with PK deficiency have been reported;^6^ its association with CML has not been reported. Our patient had shown triple mutation viz. heterozygous mutation in PKLR gene, heterozygous deletion in alpha thalassemia mutation, and BCR-ABL gene rearrangement with hemolytic anemia and CML phenotypically. The finding of all three mutations in the same patient, which has never been recorded, makes this report warranted.

The patient was managed with tyrosine kinase inhibitors for CML and component support to maintain the Hb >8 g/dL. Mitapivat, an allosteric activator of PK is FDA approved treatment of PK deficiency ^7^ and in a study by Kuo et al. it showed positive effects in reducing anemia, hemolysis, and ineffective erythropoiesis in patients with α- and β-thalassemia.^8^^,^^9^ However, it was not used in this case due to resource constraints.

The abnormalities were detected by mutational analysis studies and next generation clinical exome sequencing. These advanced diagnostic tools have enabled the detection of rare conditions. Perhaps, many mutational abnormalities might be detected if undiagnosed hematological disorders are subjected to these advanced diagnostic modalities.

In conclusion, to our knowledge, this is the first description reporting PK deficiency, heterozygous deletion as alpha thalassemia trait, and chronic myeloid leukemia in the same patient.

## Conflicts of interest

The authors declare no conflicts of interest
